# Supplementary material for: Quantized surface transport in topological Dirac semimetal films
Source: Nat Commun. 2019 Jun 12;10:2564. doi: 10.1038/s41467-019-10499-0 (PMC6561951; doi:10.1038/s41467-019-10499-0)
Supplement: Supplementary file 1 — Supplementary Information [file 41467_2019_10499_MOESM1_ESM.pdf]

Supplementary Information for
Quantized surface transport in topological Dirac semimetal films

Nishihaya *et al*.

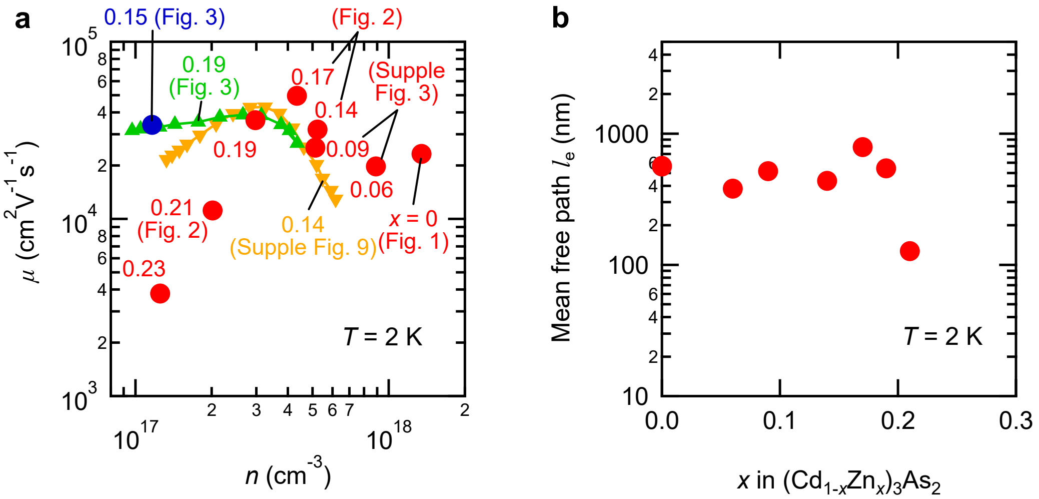

Supplementary Figure 1 | Transport properties of the $(Cd_{1-x}Zn_x)_3As_2$ films. **a**, Mobility vs electron density of $(Cd_{1-x}Zn_x)_3As_2$ films. **b**, Zn concentration dependence of mean free path l_e.

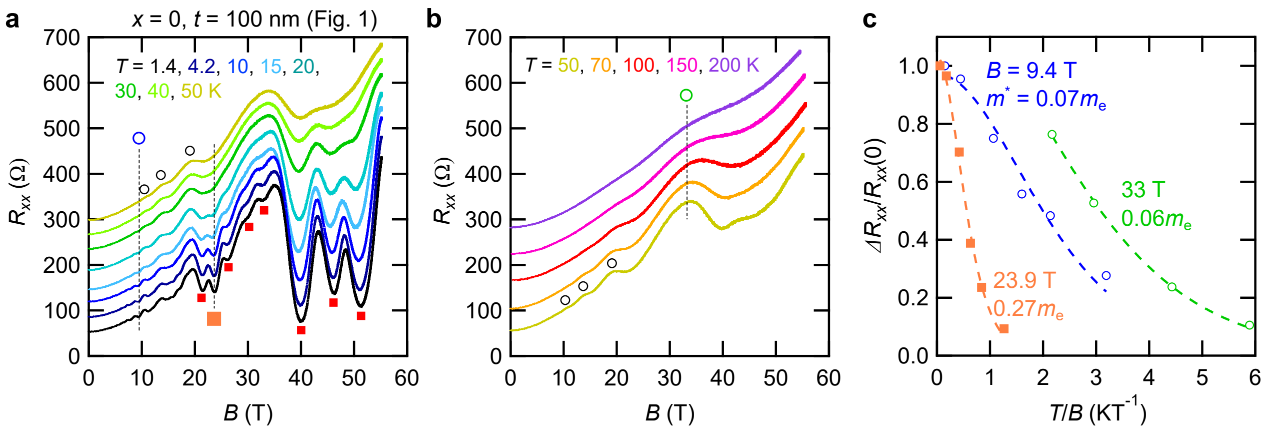

Supplementary Figure 2 | Temperature dependence of the quantum oscillations and extraction of the effective mass. a,**b**, Transverse magnetoresistance of the 100 nm thick Cd_3As_2 film at different temperatures (vertically shifted for clarity). Filled squares indicate the surface oscillations while open circles indicate the bulk oscillations. **c**, Normalized oscillation amplitudes of the three different peaks and valleys indicated by a symbol with a broken line in **a** and **b**. The broken lines in **c** are theoretical fitting curves for the extraction of the effective mass.

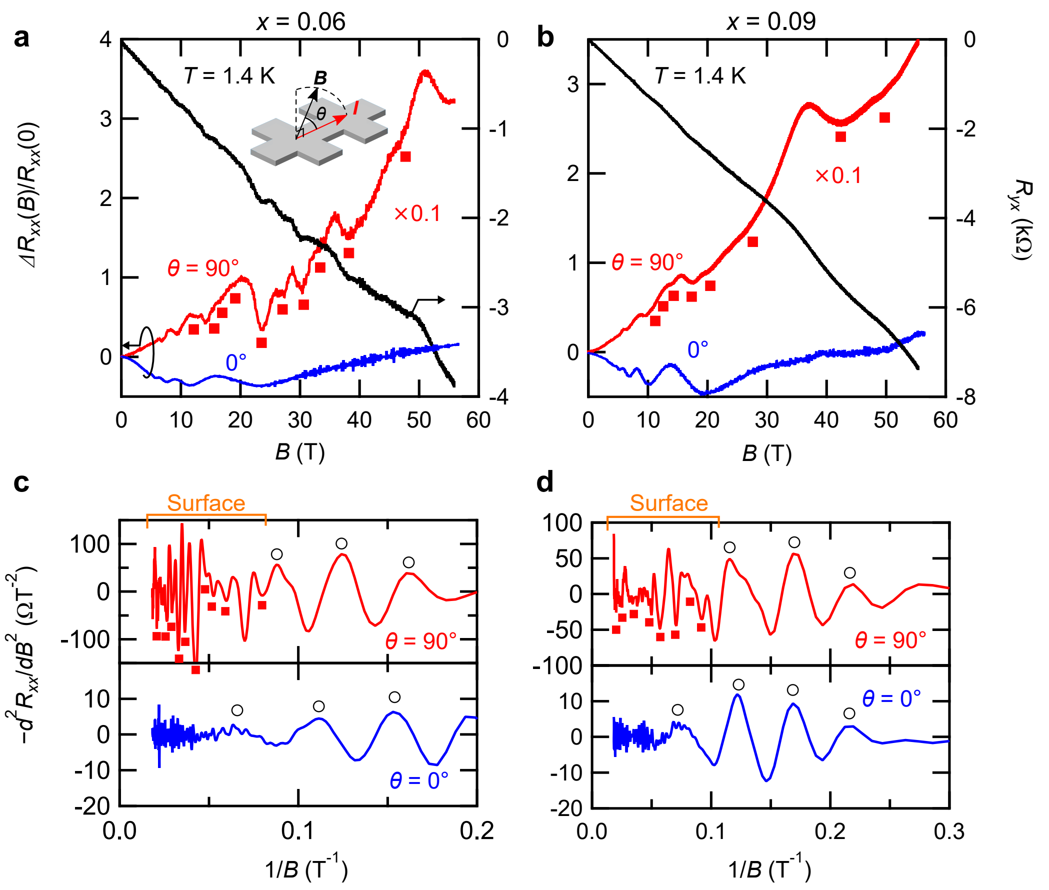

Supplementary Figure 3 | Magnetotransport of the $Cd_{1-x}Zn_x)_3As_2$ films with lower Zn concentration. Transverse and longitudinal magnetoresistances (MRs) for $x = 0.06$ (**a**) and $x = 0.09$ (**b**) measured with a pulsed field up to 55 T. Hall resistance R_{yx} is shown on the right axis. Second derivative of the MRs as a function of $1/B$ are also shown in **c** for $x = 0.06$ and in **d** for $x = 0.09$. Filled squares indicate the surface oscillations while open circles indicate the bulk oscillations.

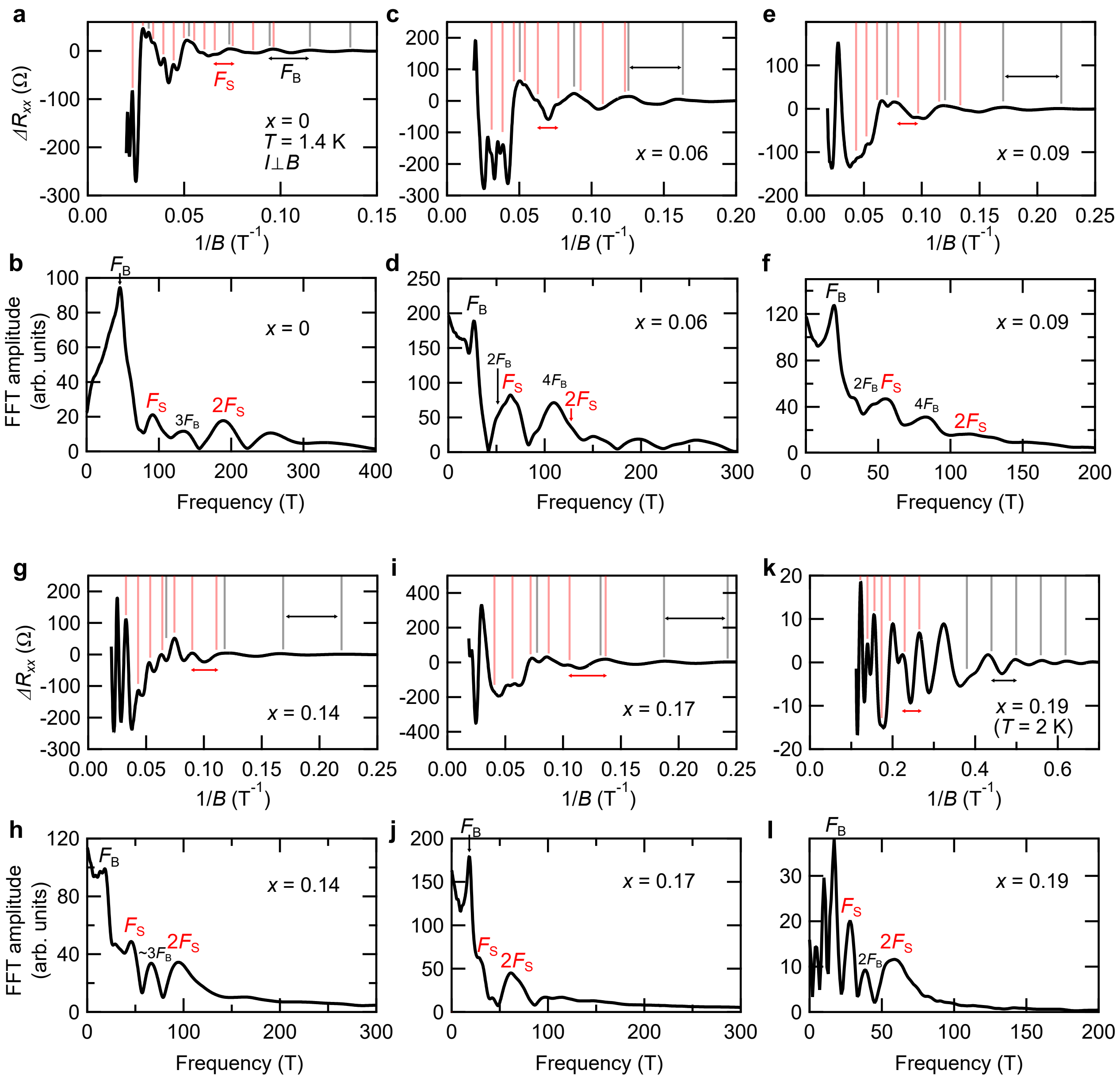

Supplementary Figure 4 | Fourier analysis of the bulk and surface quantum oscillations. Surface quantum oscillations extracted from the mangetoresistance data and their Fourier analysis results of the $(Cd_{1-x}Zn_x)_3As_2$ films (**a**,**b** for $x = 0$, **c**,**d** for 0.06, **e**,**f** for 0.09, **g**,**h** for 0.14, **i**,**j** for 0.17, and **k**,**l** for 0.19). $F_B(F_S)$ denotes the frequency of the bulk (surface) oscillations.

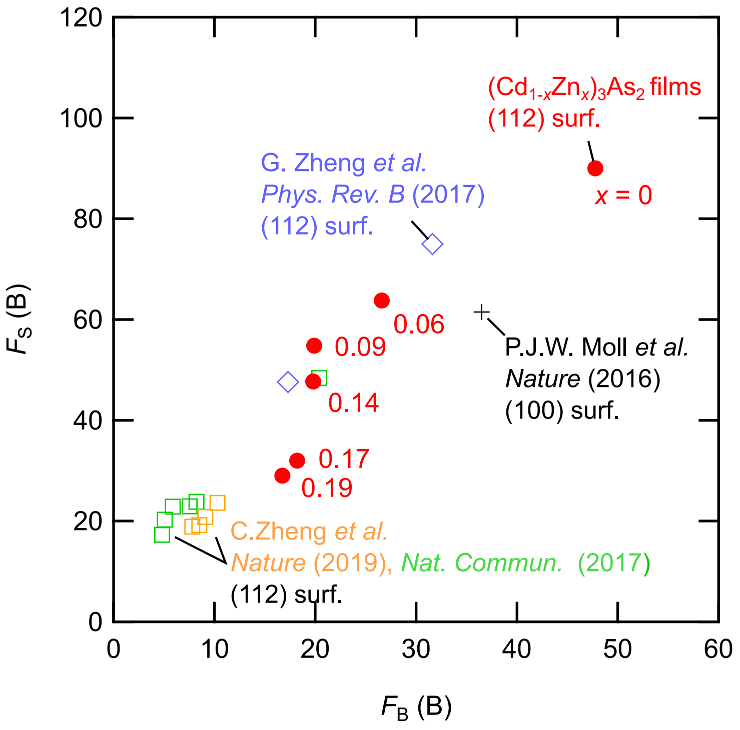

Supplementary Figure 5 | Zn concentration dependence of the surface oscillation frequency. Extracted surface oscillation frequency (F_S) as a function of bulk oscillation frequency (F_B) for the $(Cd_{1-x}Zn_x)_3As_2$ films. Data on bulk Cd_3As_2 taken from literature[6–9] are also plotted.

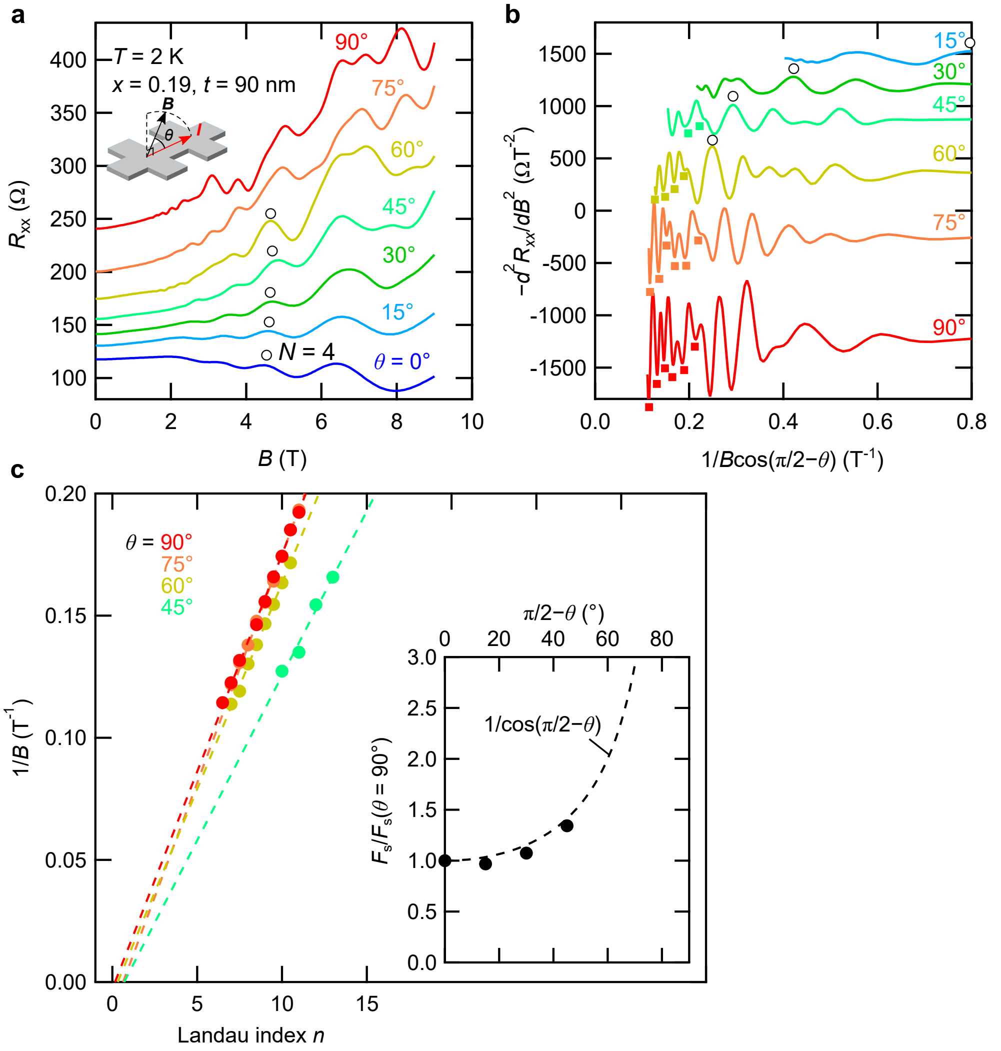
a
T = 2 K
x = 0.19, t = 90 nm
B
θ
I
90°
75°
60°
45°
30°
15°
θ = 0°
N = 4
R_{xx} (Ω)
B (T)
b
15°
30°
45°
60°
75°
90°
$-d^2R_{xx}/dB^2$ (ΩT^{-2})
1/Bcos(π/2−θ) (T^{-1})
c
θ = 90°
75°
60°
45°
1/B (T^{-1})
Landau index n
π/2−θ (°)
1/cos(π/2−θ)
$F_s/F_s(\theta = 90°)$

Supplementary Figure 6 | Field angle dependence of the surface quantum oscillations. **a**, Magnetoresistance of another $x = 0.19$ sample with a thickness of 90 nm at different field angles tilted from the current to the out-of-plane direction at 2 K. (vertically shifted for clarity). **b**, Second derivative of the magnetoresistance plotted against the inverse of the out-of-plane field component $1/B\cos(\pi/2-\theta)$ (vertically shifted for clarity). Filled squares indicate the surface oscillation valleys, while the open circles indicate the bulk $N = 4$ peak. **c**, Landau fan diagrams for the surface oscillations at different field angles. The inset shows the field angle dependence of the oscillation frequency F_{S} normalized to the $\theta = 90^{\circ}$ value.

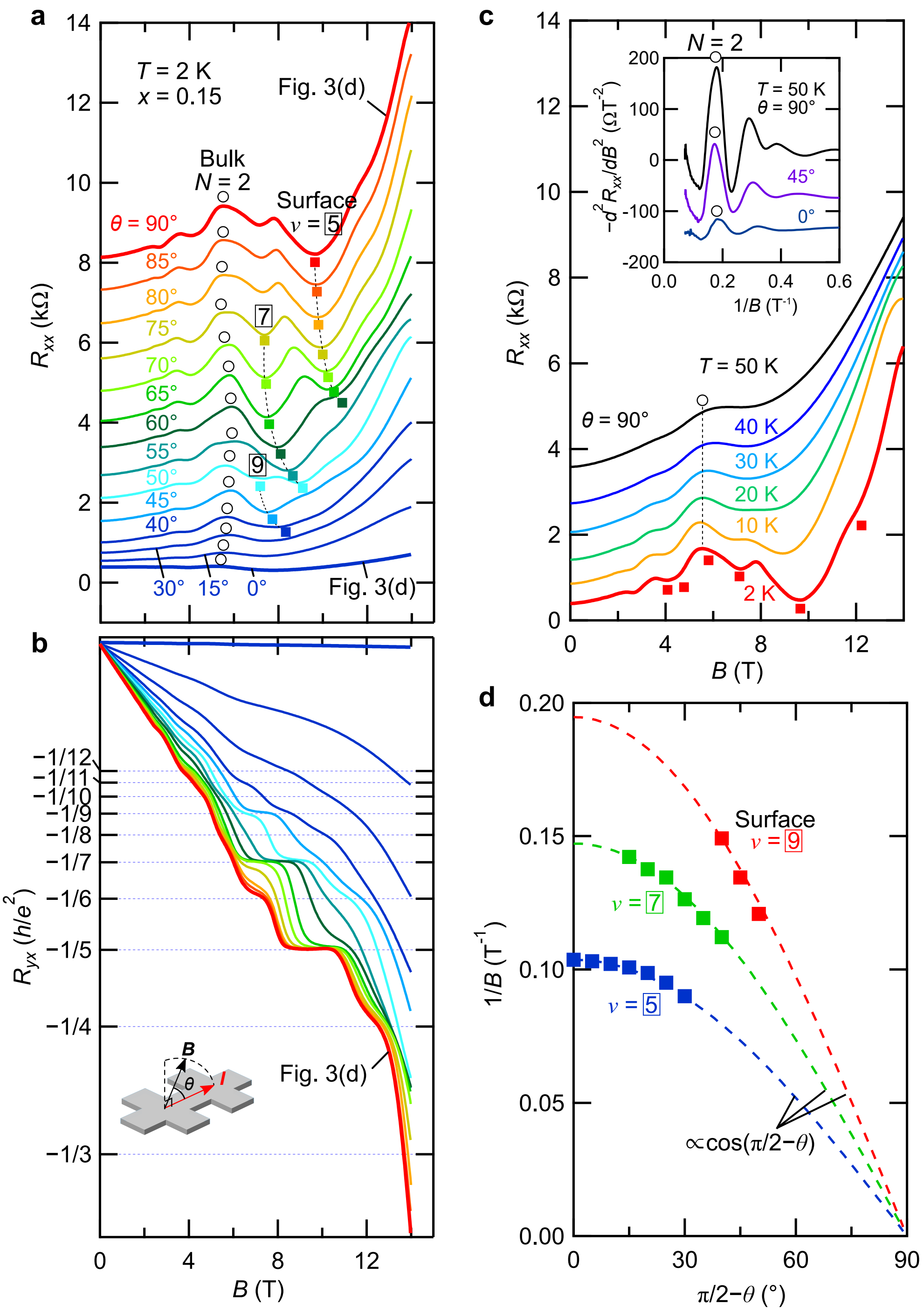

a
T = 2 K
x = 0.15
Fig. 3(d)
Bulk
N = 2
Surface
ν = 5
θ = 90°
85°
80°
75°
70°
65°
60°
55°
50°
45°
40°
30°
15°
0°
7
9
Fig. 3(d)
R_xx (kΩ)
b
R_yx (h/e^2)
−1/12
−1/11
−1/10
−1/9
−1/8
−1/7
−1/6
−1/5
−1/4
−1/3
B
θ
I
Fig. 3(d)
B (T)
c
N = 2
T = 50 K
θ = 90°
45°
0°
−d^2R_xx/dB^2 (ΩT^-2)
1/B (T^-1)
T = 50 K
θ = 90°
40 K
30 K
20 K
10 K
2 K
R_xx (kΩ)
B (T)
d
Surface
ν = 9
ν = 7
ν = 5
∝cos(π/2−θ)
1/B (T^-1)
π/2−θ (°)

Supplementary Figure 7 | Field angle dependence and temperature dependence of the surface quantum Hall states. **a**,**b**, Field angle dependence of the surface quantum Hall states in the $x = 0.15$ sample at 2 K. Filled squares indicate the quantum Hall plateaus, while open circles indicate the bulk $N = 2$ peak. **c**, Temperature dependence of the quantum Hall states at $\theta = 90°$. The inset presents the field angle dependence of the oscillation component at 50 K. **d**, Field angle dependence of the quantum Hall plateaus ($\nu = 5$, 7, and 9) at 2 K.

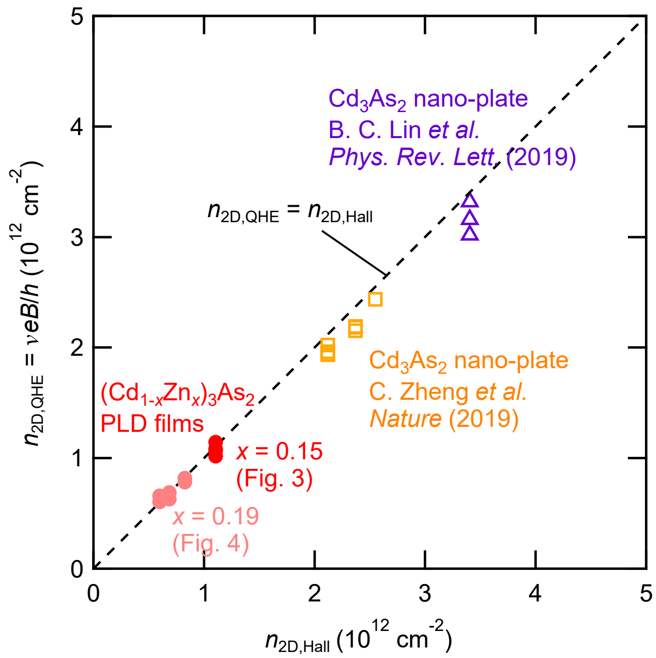

Supplementary Figure 8 | Carrier density analysis of the surface quantum Hall states. Electron density involved in the surface quantum Hall states ($n_{\mathrm{2D,QHE}}$) plotted against the total sheet density ($n_{\mathrm{2D,Hall}}$) obtained from the low-field Hall measurement. The data for $x = 0.15$ (Fig. 3) and $x = 0.19$ ($V_{\mathrm{G}} = -8$, -9, and -10 V in Fig. 4) is presented together with Cd_3As_2 nano-plate data from previous studies[9,10].

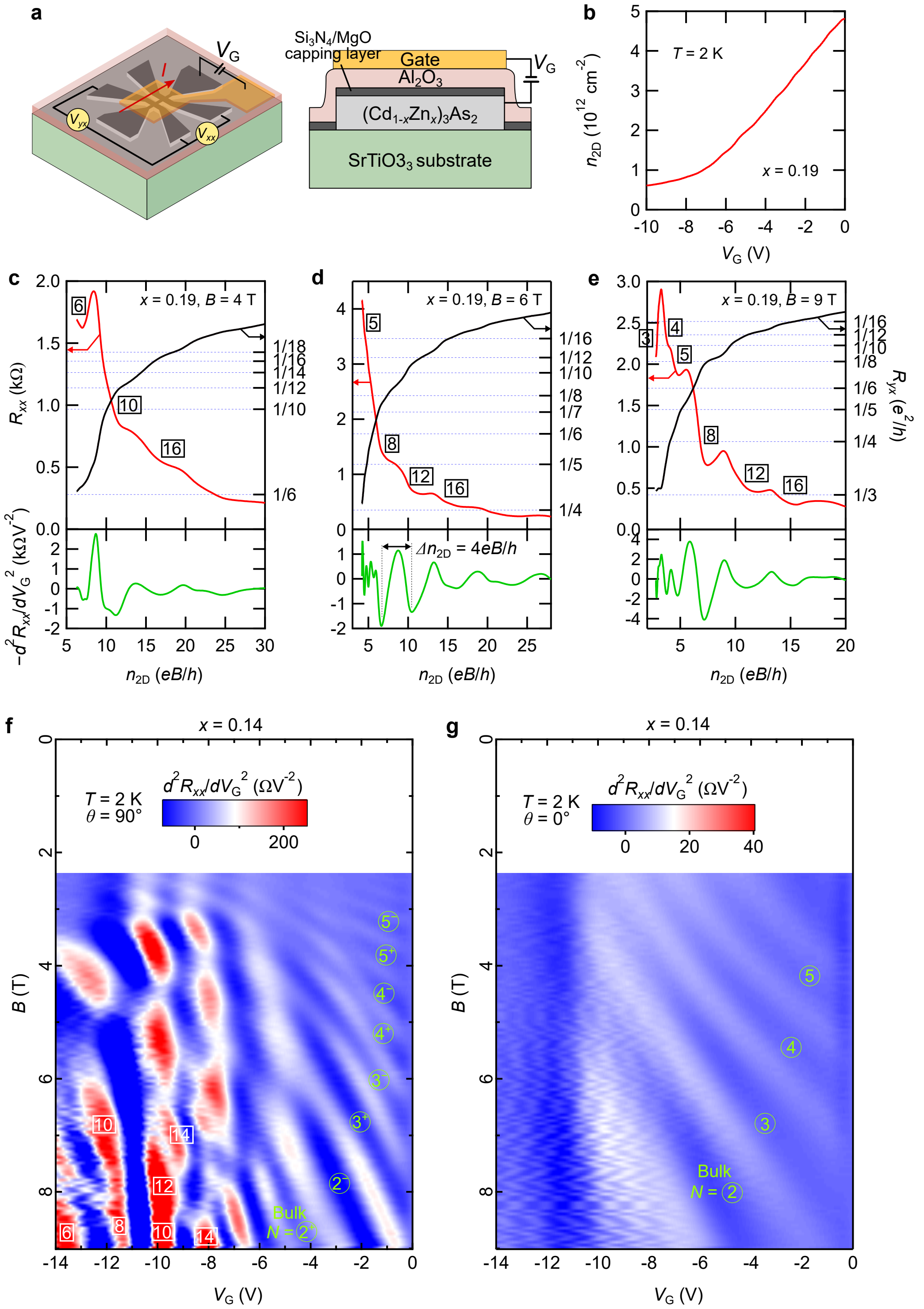

a
b
c
d
e
f
g
Si3N4/MgO capping layer
Gate
Al2O3
(Cd1-xZnx)3As2
SrTiO3 substrate
VG
T = 2 K
x = 0.19
VG (V)
x = 0.19, B = 4 T
x = 0.19, B = 6 T
x = 0.19, B = 9 T
Δn2D = 4eB/h
n2D (eB/h)
x = 0.14
T = 2 K
θ = 90°
θ = 0°
Bulk
B (T)

Supplementary Figure 9 | Gate-modulation of the electron density and the surface quantum Hall states. **a**, Top-gating configuration adopted for electrostatic gating of the $(Cd_{1-x}Zn_x)_3As_2$ films. **b**, Gate-voltage V_G dependence of sheet carrier density n_{2D} for the $x = 0.19$ sample. Longitudinal resistance R_{xx}, Hall resistance R_{yx} (upper panel) and second derivative of R_{xx} (lower panel) plotted against n_{2D} for $B = 4$ (**c**), 6 (**d**), and 9 T (**e**). The bottom axis n_{2D} is shown in the unit of eB/h so that the change in n_{2D} provides an estimate of the change in the filling factor ν. Contour mappings of the second derivative of transverse (**f**) and longitudinal (**g**) magnetoresistances as a function of V_G and field B for a $x = 0.14$ sample.

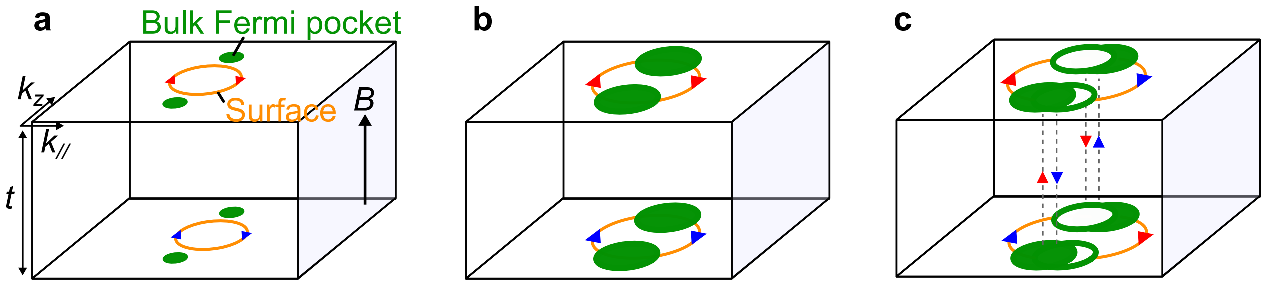

Supplementary Figure 10 | Possible surface magnetic orbits in $(Cd_{1-x}Zn_x)_3As_2$ films. **a**, Conventional magnetic orbit localized on each slab surface, which is formed by the deformation of the two Fermi-arcs into a closed Fermi contour. **b**,**c**, Magnetic orbits formed by jointed bulk and surface states. While the non-chiral bulk Landau Levels connect the two Fermi-arcs on the same surface in **b**, the chiral $N = 0$ bulk level connects the two Fermi-arcs on the opposite surfaces by transferring charges across the slab thickness t as shown in **c**.

Supplementary Note 1 | Mobility, electron density, and mean free path of the $(Cd_{1-x}Zn_x)_3As_2$ films.

$(Cd_{1-x}Zn_x)_3As_2$ (112) films were grown by the combination of pulsed laser deposition and subsequent thermal annealing. The detailed procedure and resulting crystalline quality of the film samples are reported in our previous papers[1–3]. Supplementary Fig. 1a summarizes electron mobility μ as a function of density n of the $(Cd_{1-x}Zn_x)_3As_2$ films. The results of electrostatic gating for the $x = 0.14$ and $x = 0.19$ samples are also plotted (green and yellow triangles, respectively). Increasing the Zn concentration over $x = 0.19$ leads to a systematic decrease in the mobility. Supplementary Fig. 1b shows the mean free path calculated by using the following formula; $l_e = v_F\tau = \mu m^* v_F/e$. e is the elementary charge and the values of Fermi velocity v_F and effective mass m^* of the $(Cd_{1-x}Zn_x)_3As_2$ films were previously reported in Ref. 3, where both m^* and v_F show an overall decreasing tendency upon increasing the Zn concentration. For all the $x \leq 0.21$ samples, the mean free path exceeds the film thickness (85∼100 nm), meeting the requirement for observing the unconventional magnetic orbit formed across the bulk thickness in the Dirac semimetal phase.

Supplementary Note 2 | Extraction of the effective mass from the temperature dependence of the quantum oscillations.

The effective mass m^* of the bulk and surface states in Fig. 1e was extracted from the temperature dependence of the quantum oscillations using the Lifshitz-Kosevich formula[4]. Supplementary Figs. 2a and 2b summarize the temperature dependence of the transverse magnetoresistance of the 100 nm thick Cd_3As_2 film presented in Fig. 1. The oscillation component from the surface state decays quickly with increasing the temperature, and the oscillations from the bulk state become more evident above 50 K. A similar tendency has been also reported for (112) bulk nano-plates in previous studies[7,8] To extract the oscillation amplitude, the non-oscillating background determined by the oscillation nodes ($d^2R_{xx}/dB^2 = 0$)[5] was first subtracted from the raw data. Because the surface quantum oscillations are typically superimposed on those of the bulk, the bulk component was treated as a background exceptionally when extracting the surface oscillation amplitude. The extracted oscillation amplitude and its temperature dependence are presented in Fig. 1d. m^* is obtained by fitting the temperature dependence of the normalized oscillation amplitude $\Delta R_{xx}/R_{xx}(0)$ with the function $\chi/\sinh(\chi)$. Here, $R_{xx}(0)$ is the zero-field resistance, and $\chi = 2\pi^2 m^* k_{\mathrm{B}} T/e\hbar B$, where $\hbar$ is the reduced Planck constant and k_{B} the Boltzmann constant. Supplementary Fig. 2c presents the fitting of oscillation amplitudes for representative peaks and valleys indicated by a symbol with a vertical broken line in Supplementary Figs. 2a and 2b. The larger m^* is ascribed to the surface state (filled square), while the smaller ones originate from the bulk state (open circles).

Supplementary Note 3 | Fourier analysis of the surface quantum oscillations in the $(Cd_{1-x}Zn_x)_3As_2$ films

Supplementary Fig. 3 summarizes out-of-plane transverse ($\theta = 90°$) and in-plane longitudinal magnetoresistances (MRs) ($\theta = 0°$) for $x = 0.06$ (3a) and $x = 0.09$ (3b). θ is the angle between magnetic field B and current I. As clearly seen in the second derivative of the MRs (Supplementary Figs. 3c and 3d), an oscillation component with a higher frequency than that of the bulk is observed in the transverse MR for both the samples, indicating the contribution from the surface state of the Dirac semimetal phase. The Fourier analysis results of the surface and bulk oscillations observed for $x \leq 0.19$ are presented in Supplementary Fig. 4. The high field range with well developed quantum Hall states is avoided for the Fourier transformation. The surface oscillation frequency F_S is well resolved from the bulk oscillation frequency F_B. We note that an apparent splitting of surface quantum oscillations due to degeneracy lifting is observed in higher fields, resulting in the appearance of higher harmonic peaks. Supplementary Fig. 5 presents F_S vs. F_B for the $(Cd_{1-x}Zn_x)_3As_2$ films together with bulk data in previous studies[6–9]. The F_S/F_B ratio of the non-doped (112) film is on the same trend as the previous (112) surface data taken on Cd_3As_2 bulk nano-plates. The relative shrink of F_S compared to F_B with increasing the Zn concentration x is also consistent with the picture of Zn-doping-induced topological phase transition.

Supplementary Note 4 | Field angle dependence of the surface quantum oscillation.

Supplementary Fig. 6a summarizes bulk and surface quantum oscillations of another $x = 0.19$ sample with different thickness from the one shown in Fig. 4. Supplementary Fig. 6a presents magnetoresistance curves with the magnetic field tilted from the current direction to the out-of-plane direction, and Supplementary Fig. 6b shows their second derivative. As the out-of-plane field component $B\cos(\pi/2-\theta)$ increases, quantum oscillations from the surface state emerge, complicating the oscillation patterns. Supplementary Fig. 6c presents the Landau fan diagrams of the surface oscillation peaks and valleys at different field angles. The surface oscillation frequency F_{S} extracted by a linear fit exhibits the $1/\cos(\pi/2-\theta)$ dependence (inset of 6c), consistent with the two-dimensional nature of the surface transport.

Supplementary Note 5 | Field angle dependence and temperature dependence of the surface quantum Hall states.

Supplementary Figs. 7a and 7b summarize field angle dependence of the surface quantum Hall states in the $x = 0.15$ sample at 2 K (shifted vertically for clarity). The quantum Hall states develop around the bulk oscillation valley and the strict quantization of Hall resistance R_{yx} can be observed from $\theta = 90^\circ$ down to 40°. Due to the larger effective mass of the surface state, the surface quantum Hall states quickly fade away with increasing temperature (Supplementary Fig. 7c). At 50 K, only the quantum oscillations from the bulk Fermi surface are observed as

indicated by their field angle dependence in the inset. Supplementary Fig. 7d presents the field angle dependence of the quantum Hall plateau positions which follows the $1/\cos(\pi/2-\theta)$ curve expected for the two dimensional quantized states.

Supplementary Note 6 | Carrier density analysis of the surface quantum Hall states.

Supplementary Fig. 8 presents the carrier density involved in the surface quantum Hall states $n_{\mathrm{2D,QHE}}$ as a function of the total sheet carrier density $n_{\mathrm{2D,Hall}}$ for the $(Cd_{1-x}Zn_x)_3As_2$ films as well as Cd_3As_2 bulk nano-plates in previous studies[9,10]. $n_{\mathrm{2D,QHE}}$ is calculated from the field position B and the filling factor ν of each quantum Hall state according to the relation $n_{\mathrm{2D,QHE}} = \nu eB/h$ (e is the elementary charge and h is the Planck constant). $n_{\mathrm{2D,Hall}}$ is obtained from the low field Hall measurement. Interestingly, $n_{\mathrm{2D,QHE}}$ almost equals to $n_{\mathrm{2D,Hall}}$ for all the samples showing well developed quantum Hall plateaus. $n_{\mathrm{2D,Hall}}$ should include not only the electrons occupying the surface states but also those in the bulk states. Therefore, the commonly observed relation of $n_{\mathrm{2D,QHE}} = n_{\mathrm{2D,Hall}}$ in the surface quantum Hall states indicate that all sheet carriers including those in bulk states are involved in the two-dimensional quantized transport. While a naive interpretation for such an observation would be carrier redistribution possibly induced by the unique magnetic orbits weaving the bulk and surface states together, further investigations are required to clarify the details of quantization mechanism of the emergent surface transport. We also note that because the quantization fields are determined by the total sheet carrier density ($1/B = \nu e/n_{\mathrm{2D,Hall}}h = \nu e/tn_{\mathrm{3D,Hall}}h$, t is the film thickness and $n_{\mathrm{3D,Hall}}$ is the density per volume), the surface quantum

Hall states may not be explained simply by extending the semiclassical formula originally proposed for Weyl orbit quantum oscillations[11] ($1/B = \frac{4\pi^2 e}{hF_S}(n + \gamma - t\frac{E_F}{\pi v})$, where F_S is the area enclosed by the surface states, n the Landau index, γ the phase correction term, E_F the Fermi level, and v the Fermi velocity along the field) to the quantized transport.

Supplementary Note 7 | Gate-modulation of the electron density and the surface quantum Hall states.

The electrostatic depletion of the electron density in $(Cd_{1-x}Zn_x)_3As_2$ films was conducted by adopting a top-gate configuration (Supplementary Fig. 9a). Supplementary Fig. 9b shows V_G dependence of the sheet carrier density n_{2D}. In quantum Hall states, each Landau level has a degeneracy of eB/h, and thus, the change $\Delta\nu$ in the filling factor ν when sweeping V_G at a fixed field corresponds to a change of the sheet electron density by $\Delta\nu eB/h$. In Supplementary Figs. 9c-9e, the resistance R_{xx} and its second derivative are presented as a function of n_{2D} for representative magnetic fields (B = 4, 6, and 9 T). Hall resistance R_{yx} is also shown on the right axis. While the filling factors at each V_G scan in Fig. 4 are mainly assigned by referring to the values of R_{yx}, the assignment can also be verified from the change in n_{2D} between neighboring oscillation valleys.

Supplementary Figs. 9f and 9g show the V_G-B contour mapping of the quantum oscillations for a $x = 0.14$ sample. Similarly to the $x = 0.19$ case presented in Fig. 4, the contribution of the surface quantum transport starts to dominate the transverse magnetoresistance upon decreasing electron density (Supplementary Fig. 9f). The characteristic four-fold degeneracy of the quantum

Hall states can also be observed. Due to the decreased mobility by gating (Supplementary Fig. 1a), the quantization of R_{yx} are too weak to allow a precise determination of the filling factors in lower fields.

Supplementary Note 8 | Possible surface magnetic orbits in $(\mathbf{Cd}_{1-x}\mathbf{Zn}_x)_3\mathbf{As}_2$ films.

To produce quantum oscillations from the Fermi-arc state under magnetic fields, two Fermi-arc states need to be interconnected to complete a closed magnetic orbit. A Dirac semimetal (DSM) consists of two copies of a Weyl semimetal (WSM) with opposite chirality, which are protected from mixing by the crystal rotational symmetry. On each surface of a DSM slab, there exist two Fermi arcs curving in opposite directions and meeting at bulk Weyl points (or bulk Fermi pockets). Therefore, unlike the case of WSM, there are several possible ways for the Fermi-arcs in DSM to form a closed orbit under magnetic fields.

One is that the double Fermi-arcs on the same surface deform into a closed Fermi contour to form a conventional orbit. Kargarian *et al.* have reported that the double Fermi-arcs are not topologically protected and a perturbation leading to the Fermi-arcs deformation can exist in general without gapping the bulk nodes[12]. When the Fermi level is low, the Fermi contour no longer merges with the bulk states, giving rise to magnetic orbits which are independently localized on each slab surface (Supplementary Fig. 10a). When the Fermi level is high, on the other hand, the bulk Fermi pockets start to merge with the Fermi contour, and the situation becomes similar to the case where the double Fermi-arcs terminate at the bulk Fermi pockets. Then, the closed magnetic

orbits are formed not only within the surface states but rather via the bulk states connecting the two fermi-arcs. In DSM, there are two kinds of bulk states under the fields; a chiral $N = 0$ Landau level (LL) and non-chiral $N \neq 0$ LLs[13]. The chiral $N = 0$ LL can transfer electrons from one slab surface to the other across the slab thickness, while the non-chiral LLs cannot. Therefore, there are two possible patterns in the bulk-involved magnetic orbits; one is a conventional orbit formed by two Fermi-arcs on the same slab surface (Supplementary Fig. 10b) similar to the topological insulator case (Fig. 1c), and the other one is the Weyl orbit with two Fermi-arcs on the opposite slab surfaces connected via the bulk $N = 0$ LL (Supplementary Fig. 10c)

As shown in Fig. 4b (x = 0.19) and Supplementary Fig. 9f (x = 0.14), a four-fold degeneracy has been observed in the surface quantum states during the electrostatic gating. The observed degeneracy cannot be explained by the conventional surface orbits depicted in Supplementary Fig. 10a, where the degeneracy is only 2. Taking into account the number of occupied bulk Landau states, the four-fold degeneracy suggests the involvement of the bulk states in the formation of the surface magnetic orbits, and possible coexistence of two different kinds of orbits. At this point, however, whether the Weyl orbits are realized via the bulk $N = 0$ modes is not clear. At large gate-voltage V_G or at high fields, the two-fold degeneracy of the remaining $N = 0$ orbits eventually reduced to 1. This Zeeman-splitting-like degeneracy lifting from 2 to 1 has been also confirmed in other $(Cd_{1-x}Zn_x)_3As_2$ films under pulsed fields up to 55 T (such as shown in Fig. 1d), as well as in low-carrier density bulk nano-plates in previous studies[9,10]. While a naive expectation would be that the Weyl orbits in DSM should be always doubly degenerate due to the preserved inversion

symmetry and lifting of the two-fold degeneracy seems to contradict the Weyl orbit scenario, we note that the effect of high field application on the DSM can be complicated due to the competition between rotational symmetry (RS) breaking and time-reversal symmetry (TRS) breaking effects. Depending on which effect dominates, there are several possibilities where the paired Weyl orbits in DSM can exhibit a degeneracy lifting as discussed below.

One is a RS-breaking-induced transition from the Weyl orbits (Supplementary Fig. 10c) to the conventional surface orbits (Supplementary Fig. 10b) in a high-field or low-density regime. In fact, the field configuration for realizing the Weyl orbits in DSM always breaks its RS leading to a gap Δ_B in the bulk chiral modes. It has been theoretically discussed that when the energy gap Δ_B becomes comparable to the Fermi level $E_{\rm F}$, the electrons cannot tunnel across the bulk anymore[11]. The resulting conventional surface orbits (Supplementary Fig. 10b) can show lifting of the two-fold degeneracy due to any asymmetric conditions between the top and bottom surfaces, such as induced by gating or interfaces in film samples. We also note that application of strong electric field on one surface may also induce a similar transition from unconventional to conventional surface orbits especially when $E_{\rm F}$ is low due to the asymmetric Weyl points shift between the two surfaces.

Another possibility is the TRS breaking induced phase transition from DSM to WSM. If the exchange energy between the field and the bulk orbitals dominates Δ_B, the DSM phase is first driven into TRS broken WSM[14]. In this case, the RS is no longer necessary to protect the bulk chiral mode, and the Weyl orbits (Supplementary Fig. 10c) can be maintained. The topological

phase transition process depends on the magnetic field strength and field direction and can result in an asymmetric change of the Fermi-arc length between the two Weyl orbits, lifting the two-fold degeneracy. Again, this degeneracy lifting effect may be further enhanced by gating or interface asymmetry in the case of film samples. Therefore, the observed two-fold degeneracy lifting does not exclude the Weyl orbit scenario, though further investigations are required to unambigiously identify the detailed surface orbits and their underlying quantization mechanism in DSM.

Supplementary References

1. Uchida, M. *et al*. Quantum Hall states observed in thin films of Dirac semimetal Cd_3As_2. *Nat. Commun.* **8,** 2274 (2017).

2. Nakazawa, Y. *et al*. Structural characterisation of high-mobility Cd_3As_2 films crystallised on $SrTiO_3$. *Sci. Rep.* **8,** 2244 (2018).

3. Nishihaya, S. *et al*. Negative magnetoresistance suppressed through a topological phase transition in $(Cd_{1-x}Zn_x)_3As_2$ thin films. *Phys. Rev. B* **97,** 245103 (2018).

4. Shoenberg D., *Magnetic Oscillations in Metals* (Cambridge University Press, Cambridge, U.K., 1984).

5. Murakawa, H. *et al*. Detection of Berry's phase in a bulk Rashba semiconductor. *Science* **342,** 1490-1493 (2013).

6. Moll, P. J. W. *et al*. Transport evidence for Fermi-arc-mediated chirality transfer in the Dirac semimetal Cd_3As_2. *Nature* **535,** 266-270 (2016).

7. Zhang, C. *et al*. Evolution of Weyl orbit and quantum Hall effect in Dirac semimetal Cd_3As_2. *Nat. Commun.* **8,** 1272 (2017).

8. Zheng, G. *et al*. Recognition of Fermi-arc states through the magnetoresistance quantum oscillations in Dirac semimetal Cd_3As_2 nanoplates. *Phys. Rev. B* **96,** 121407(R) (2017).

9. Zhang, C. *et al*. Quantum Hall effect based on Weyl orbits in Cd_3As_2. *Nature* **565,** 331-336 (2019).

10. Lin, B.-C. *et al*. Observation of an odd-integer quantum Hall effect from topological surface states in Cd_3As_2. *Phys. Rev. Lett.* **122,** 036602 (2019).

11. Potter A. C., Kimchi, I. & Vishwanath, A. Quantum oscillations from surface Fermi arcs in Weyl and Dirac semimetals. *Nat. Commun.* **5,** 5161 (2014).

12. Kargarian M., Randeria, M. & Lu, Y.-M. Are the surface Fermi arcs in Dirac semimetals topologically protected? *Proc. Natl. Acad. Sci. USA* **113,** 8648-8652 (2016).

13. Armitage, N. P., Mele, E. J. & Vishwanath, A. Weyl and Dirac semimetals in three-dimensional solids. *Rev. Mod. Phys.* **90,** 015001 (2018).

14. Wang, Z. *et al*. Dirac semimetal and topological phase transitions in A_3Bi (A = Na, K, Rb). *Phys. Rev. B* **85,** 195320 (2012).
